# Vascular endothelial growth factor in node-positive breast cancer patients treated with adjuvant tamoxifen

**DOI:** 10.1038/sj.bjc.6601060

**Published:** 2003-07-15

**Authors:** D Coradini, E Biganzoli, C Pellizzaro, S Veneroni, S Oriana, F Ambrogi, R Erdas, P Boracchi, M G Daidone, E Marubini

**Affiliations:** 1 Unità Operativa Determinanti Biomolecolari nella Prognosi e Terapia, Istituto Nazionale per lo Studio e la Cura dei Tumori, Via Venezian, 1, 20133 Milan, Italy; 2 Unità di Statistica Medica e Biometria, Istituto Nazionale per lo Studio e la Cura dei Tumori, Via Venezian, 1, 20133 Milan, Italy; 3 Unità di Chirurgia Senologica, Istituto Nazionale per lo Studio e la Cura dei Tumori, Via Venezian, 1, 20133 Milan, Italy; 4 Istituto di Statistica Medica e Biometria, Università degli Studi di Milano, 20133 Milan, Italy

**Keywords:** vascular endothelial growth factor, steroid receptors, node-positive breast cancer, tamoxifen

## Abstract

In 212 postmenopausal women with node-positive oestrogen receptor-positive (ER_LBA_) breast cancer subjected to radical surgery and adjuvant tamoxifen, the risk of 6-year relapse increased with increasing values of intratumoral vascular endothelial growth factor (VEGF) in patients whose tumours had a low/intermediate ER_LBA_ content compared to patients with high-ER_LBA_ tumours. These findings indicate that tumour progression, activated or sustained by high VEGF levels, may be counteracted in high-ER_LBA_ cancers by tamoxifen, which in contrast fails to contrast the metastatic potential in low-ER_LBA_ tumours.

Breast tissue is highly responsive to changes in ovarian hormone concentrations that induce a cyclic remodelling process involving epithelial, stromal and vascular components during each menstrual cycle (Vogel *et al*, 1981). In particular, the angiogenic turnover is regulated by oestrogens and progesterone, which modulate the expression of vascular endothelial growth factor (VEGF) in the epithelial cells of the terminal ductal–lobular units (Nakamura *et al*, 1999).

Although the VEGF gene promoter lacks a perfect palindromic oestrogen-responsive element, an analogue of the consensus element that functions as a classical enhancer for oestrogen receptor (ER) has been identified in the 3′-untranslated region (Hyder *et al*, 2000b).

Angiogenesis is known to represent a fundamental step in tumour progression (Hanahan and Folkman, 1996) and clinical evidence has shown that node-negative (N−) as well as node-positive (N+) breast cancer patients with high intratumoral VEGF concentrations have a significantly shorter relapse-free survival (RFS) (Gasparini *et al*, 1997, 1999; Linderholm *et al*, 2000).

Preclinical studies on breast cancer cell lines demonstrated that, as expected, oestrogens rapidly induce VEGF expression, which is blocked by pure oestrogen antagonists (Ruohola *et al*, 1999; Hyder *et al*, 2000a).

In a series of N+ ER-positive (ER+, by ligand-binding assay, LBA) postmenopausal women with resectable breast cancer who received adjuvant tamoxifen, we investigated the comprehensive effect on RFS of VEGF content and steroid receptor profile, evaluated in the same cytosolic fraction.

## PATIENTS AND METHODS

### Patients

The study included postmenopausal patients with primary resectable invasive breast cancer, histologically classified as N+, who underwent surgery at the Istituto Nazionale Tumori in Milan between March 1991 and December 1995 and received only adjuvant tamoxifen (20 mg day^−1^) for at least 2 years (median duration time, 4 years) because of their positive ER status (ER tumour concentration higher than 10 fmol mg^−1^ of protein). From a total of 859 N+, ER+ postmenopausal patients, consecutive with respect to steroid receptor determination at the time of diagnosis, 289 were selected on the basis of treatment, histology (pure or mixed ductal or lobular invasive tumours) and follow-up (i.e. a minimum potential of 6 years from the date of surgery to the date of last updating of patient records).

Of the 289 eligible patients, 212 (73%) were available for VEGF evaluation. Their median age was 64 years (range, 50–85); 88 patients (42%) were treated by mastectomy and 124 (58%) by breast-conserving surgery plus radiotherapy. All of them underwent complete axillary lymph node dissection (median number of examined nodes, 18). Most patients had one to three metastatic axillary lymph nodes (139, 66%). Small (⩽2 cm) and large (>2 cm) tumours were equally represented (93, 48% and 99, 52%, respectively). After surgery, the patients were followed at 6-month intervals during the first 5 years and at 12-month intervals thereafter; disease status was assessed by means of physical examination, chest X-ray, bone scan and abdominal sonography. Treatment failure was defined as the first documented evidence of new disease manifestations in locoregional areas (seven cases), distant sites (40 cases), or in the contralateral breast (five cases). Relapse-free survival was calculated as the time elapsed from diagnosis to the date of first recurrence or to the last clinical examination for patients without documented disease manifestation. Median follow-up for the whole series was 68 months (interquartile range, 52–82 months).

### Steroid receptor determination by LBA

Steroid receptor content was determined according to the EORTC recommendations and within national (Piffanelli *et al*, 1991) and international (EORTC Breast cancer co-operative group, 1980) quality control programmes by a double-labelling assay (Coradini *et al*, 2000), and expressed as fmol mg^−1^ of protein. Tumours with an ER_LBA_ concentration higher than 10 fmol mg^−1^ of protein were defined as ER+.

### Vascular endothelial growth factor determination

The predominant VEGF isoform, VEGF_65_ (henceforward referred to as VEGF), was measured by a quantitative enzyme immunoassay technique (Quantikine, human VEGF; R&D Systems, Minneapolis, MN, USA) as described elsewhere (Coradini *et al*, 2001). Concentrations were expressed as pg of VEGF protein per mg of total protein.

### Statistical analysis

The overall association of VEGF level with patient age, tumour size, number of metastatic lymph nodes, ER_LBA_ and PgR_LBA_ content was evaluated by Spearman's rank correlation coefficient.

The effect of VEGF, ER_LBA_ and PgR_LBA_ content on RFS was investigated by multivariate analysis using a Cox regression model in which also the number of metastatic lymph nodes was included. All variables were considered on a continuous scale after logarithmic transformation. Null values for PgR_LBA_ content were arbitrarily set at 1, taking a sensitivity threshold value of 2 fmol mg^−1^ of protein. According to a previous finding (Coradini *et al*, 2001), linear terms for log(ER_LBA_), log(PgR_LBA_) and log(VEGF) and the interaction between ER_LBA_ and VEGF were included in the model.

The proportional hazard assumption of the Cox model was evaluated and the effect of model terms was tested as previously reported (Coradini *et al*, 2001).

The library written by Harrell *et al* (1996) was applied in some steps of the model building procedure and the SAS macro programme RELIMPCR designed by Heinze and Schemper (2001) was adopted for evaluation of the relative prognostic contribution of the covariates.

## RESULTS

In this series of ER_LBA_-positive tumours from postmenopausal patients, the VEGF content ranged from 7 to 2186 pg mg^−1^ of protein with 52, 95 and 200 as the 25th, 50th and 75th percentiles, respectively; the PgR_LBA_ concentration ranged from 1 to 2563 fmol mg^−1^ of protein with 40, 111 and 355 as the 25th, 50th and 75th percentiles, respectively. Vascular endothelial growth factor content did not show any significant correlation with any of the other variables considered; estimated correlation coefficients were all in the range ±0.08.

In the multivariate model, the number of metastatic lymph nodes, VEGF, ER_LBA_ and the interaction between ER_LBA_ and VEGF were significantly related to prognosis (Table 1

**Table 1:** Final Cox regression model results for RFS

**Effect**	**Wald statistics**	**d.f.**	**b**^a^	***P***
Metastatic nodes	19.76	1	0.6054	<0.0001
VEGF	7.70^b^	2	1.7562	0.0213
ER_LBA_	5.59^b^	2	1.5317	0.0610
PgR_LBA_	1.26	1	−0.0696	0.2617
ER_LBA_–VEGF	4.73	1	−0.3405	0.0297
				
Total	30.78	6		<0.0001

a Estimated regression coefficient.

b Factor +interaction.

), whereas no significant contribution was observed for PgR_LBA_. As for ER_LBA_, VEGF and the ER_LBA_–VEGF interaction, the results were similar to those previously obtained in patients with N– breast cancer who did not receive systemic treatment (Coradini *et al*, 2001). This finding further supports the role of ER_LBA_ in modulating the prognostic effect of VEGF and the negative interaction term indicates a decrease in the unfavourable effect of VEGF for increasing values of ER_LBA_.

**Figure 1:**
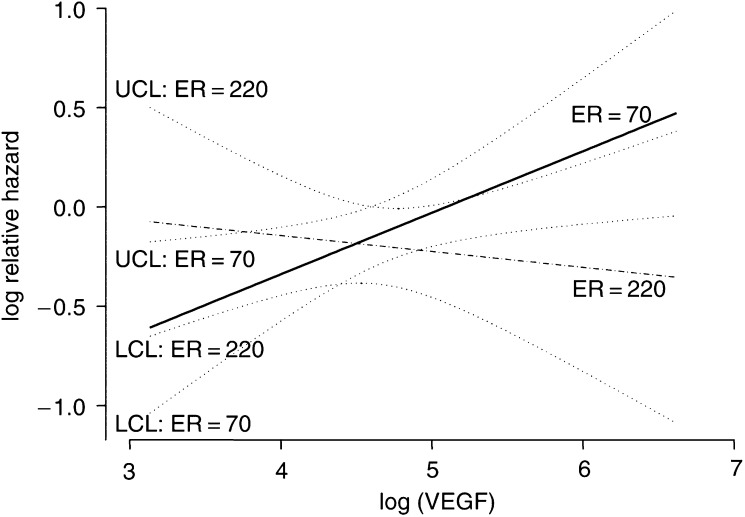
Plots of the logarithm of the relative hazard of disease recurrence as a function of VEGF level for different ER_LBA_ values (fixed approximately at the first and third quartiles of the distribution). The solid line corresponds to an ER_LBA_=70 fmol mg^−1^ protein, whereas the dashed line corresponds to an ER_LBA_=220 fmol mg^−1^ protein. Dotted lines indicate 95% pointwise confidence limits (upper: UCL; lower: LCL).

To provide a description of the combined effect of VEGF and ER_LBA_, the relative hazard (RH) for increasing VEGF concentrations was plotted (Figure 1

) for selected values of ER_LBA_ (70 and 220 fmol mg^−1^ of protein) that were approximately equal to the 1st and third quartiles of ER_LBA_ distribution. Owing to the interaction between ER_LBA_ and VEGF, when the ER_LBA_ content was set at 70 fmol mg^−1^ protein, the increase in the risk of disease recurrence for high VEGF values associated with low ER_LBA_ values was evident. In fact, the estimated RH for patients with a VEGF content of 200 *vs* those with a VEGF content of 50 pg mg^−1^ protein was 1.54 (95% CI, 1.07–2.20). Conversely, when the ER_LBA_ content was set at 220 fmol mg^−1^ protein, VEGF did not show any prognostic effect and the estimated RH for the two selected values (200 and 50 pg ml^−1^) was 0.89 (95% CI, 0.54–1.47).

Analysis of the prognostic contribution of the covariates showed that the model explained only 13.2% of the total heterogeneity of the relapse times of our case series and that, considering the relative importance of prognostic factors, 8.2% was attributable to the number of metastatic lymph nodes, whereas VEGF and ER_LBA_ together contributed with 3.3%, and PgR only with 0.8%.

## DISCUSSION

According to the recently revised treatment guidelines for early breast cancer (Goldhirsch *et al*, 2001), postmenopausal women with ER+ primary breast cancer should receive adjuvant hormone therapy with tamoxifen. However, a recent clinical study (Linderholm *et al*, 2000) provided evidence that also the intratumoral VEGF content could predict outcome following adjuvant endocrine treatment: patients with ER+ tumours, but a high VEGF expression, had a significantly shorter RFS and overall survival. In agreement with these findings, our results showed that, due to the presence of a negative interaction between ER_LBA_ and VEGF, already observed in a series of N− cancers (Coradini *et al*, 2001), patients whose tumours had a low/intermediate ER_LBA_ content exhibited an increased risk of disease recurrence with increasing values of intratumoral VEGF. Conversely, the VEGF level did not show prognostic effect in patients whose tumours had a high ER_LBA_ content. These findings suggest that in tumours characterised by a high ER_LBA_ concentration and therefore more likely to be hormonally regulated, tumour progression, activated or sustained by VEGF, may be counteracted by the protective effect of endocrine therapy. Conversely, when a patient has a high level of VEGF and a low ER_LBA_ concentration, tamoxifen could fail to contrast the tumour's metastatic potential and more tailored adjuvant treatment would be required including, for example, an antiangiogenic agent.
